# Production, Characterization, and Antioxidant Activity of Fucoxanthin from the Marine Diatom *Odontella aurita*

**DOI:** 10.3390/md11072667

**Published:** 2013-07-23

**Authors:** Song Xia, Ke Wang, Linglin Wan, Aifen Li, Qiang Hu, Chengwu Zhang

**Affiliations:** 1 Institute of Hydrobiology, Jinan University, Guangzhou 510632, China; E-Mails: xiasongsummer212@163.com (S.X); 476443371@qq.com (K.W.); linglinwan@jnu.edu.cn (L.W.); tiger@jnu.edu.cn (A.L.); 2 Laboratory for Algae Research and Biotechnology, College of Technology and Innovation, Arizona State University, 7001 E. Williams Field Road, Mesa, AZ 85212, USA

**Keywords:** microalgae, *Odontella aurita*, fucoxanthin, photobioreactor, antioxidant

## Abstract

The production, characterization, and antioxidant capacity of the carotenoid fucoxanthin from the marine diatom *Odontella aurita* were investigated. The results showed that low light and nitrogen-replete culture medium enhanced the biosynthesis of fucoxanthin. The maximum biomass concentration of 6.36 g L^−1^ and maximum fucoxanthin concentration of 18.47 mg g^−1^ were obtained in cultures grown in a bubble column photobioreactor (Ø 3.0 cm inner diameter), resulting in a fucoxanthin volumetric productivity of 7.96 mg L^−1^ day^−1^. A slight reduction in biomass production was observed in the scaling up of *O. aurita* culture in a flat plate photobioreactor, yet yielded a comparable fucoxanthin volumetric productivity. A rapid method was developed for extraction and purification of fucoxanthin. The purified fucoxanthin was identified as all-*trans*-fucoxanthin, which exhibited strong antioxidant properties, with the effective concentration for 50% scavenging (EC_50_) of 1,1-dihpenyl-2-picrylhydrazyl (DPPH) radical and 2,2′-Azino-bis(3-ethylbenzthiazoline-6-sulfonic acid (ABTS) radical being 0.14 and 0.03 mg mL^−1^, respectively. Our results suggested that *O*. *aurita* can be a natural source of fucoxanthin for human health and nutrition.

## 1. Introduction

Fucoxanthin is a major carotenoid in seaweeds and diatoms. This pigment forms, together with chlorophyll (Chl) *a*, Chl *c* and an apoprotein, a major light-harvesting fucoxanthin, chlorophyll *a*/*c* complex, which transfers light energy to chlorophyll *a* of the photosynthetic reaction centers for photosynthesis [1]. Fucoxanthin has an allenic bond, a conjugated carbonyl, a 5,6-monoepoxide and an acetyl groups that contribute to a unique structure of the molecule. It has been reported that this carotenoid exhibits strong antioxidant, anti-inflammatory, anti-obesity, antidiabetic, anticancer, and antihypertensive activities [2,3]. Fucoxanthin can also be used as an animal feed additive in poultry and aquaculture industries [4]. 

Owing to its broad application potential, commercial production of fucoxanthin from algae has been explored. Several studies were conducted to extract fucoxanthin from brown macroalgae, such as *Laminaria japonica*, *Eisenia bicyclis*, and *Undaria pinnatifida* [5,6]. Because these macroalgae are traditional foods in South-East Asia and some European countries, and they contain very low concentrations (e.g., 0.02 to 0.58 mg g^−1^ fresh weight) of fucoxanthin, the production of fucoxanthin from brown macroalgae is not commercially feasible [7]. Therefore, searching for alternative sources of fucoxanthin is necessary. 

Some microalgae can produce large amounts of specific carotenoids, such as β-carotene in *Dunaliella salina*, astaxanthin in *Haematococcus pluvialis*, and lutein in *Scenedesmus almeriensis* and *Muriellopsis* sp. [8]. With an estimated 100,000 species, fucoxanthin-containing diatoms constitute a large group of fresh water and marine microalgae, accounting for about 40% of the marine primary productivity and contributing to as high as 20%–25% of the global net primary production [9]. Despite their abundance and diversity in the aquatic environment, a few species have commercially been exploited for fucoxanthin production [10].

*Odontella aurita* is a bobbin-like unicell, normally ranging from 15 to 30 μm in length. The microalga contains high concentrations (28% of total fatty acids) of the long chain polyunsaturated fatty acid eicosapentaenoic acid (EPA, 20:5ω3) and has been tested for mass culture in open ponds [11]. In this study, we determined that *O*. *aurita* can accumulate high concentrations of fucoxanthin (>20 mg g^−1^ of dry weight). The effects of nitrogen concentration and light intensity on fucoxanthin production in this diatom were investigated. The commercial potential of fucoxanthin production from *O*. *aurita* was also assessed in a pilot-scale flat plate photobioreactor. A rapid extraction procedure was developed for extraction of fucoxanthin, which was further purified by a silica gel-based preparation high performance liquid chromatography (prep-HPLC). The structural characteristics and antioxidant activity of highly purified fucoxanthin were investigated. 

## 2. Results and Discussion

### 2.1. Growth Kinetics and Fucoxanthin Production in the Bubble Column Photobioreactor

Nitrogen concentration and incident light intensity are two major factors affecting growth and pigment biosynthesis of microalgae [12,13,14]. A common trend of cellular response to stress conditions, such as high light and nitrogen depletion, appears to increase secondary carotenoids (e.g., β-carotene, astaxanthin, lutein), which serve as photoprotective agents [14]. Harker *et al.* [15] reported that the astaxanthin content increased when *H*. *pluvialis* was cultivated in media deficient in nitrogen. When exposed to high light intensity, *D*. *salina* accumulated large quantities of β-carotene [16]. 

**Figure 1:**
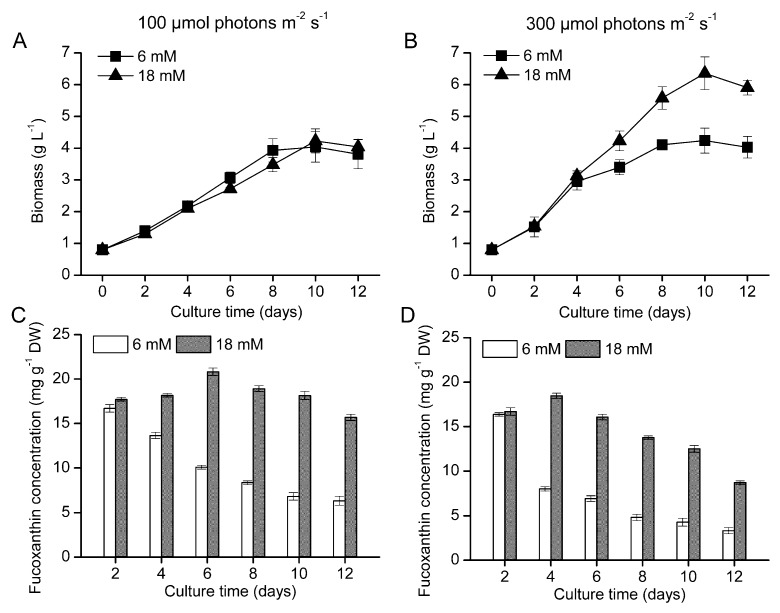
The growth profile (**A**,**B**) and fucoxanthin concentration (**C**,**D**) of *O. aurita* cultivated in the bubble column photobioreactor under 150 (**A**,**C**) and 300 (**B**,**D**) μmol photons m^−2^ s^−1^ light intensity with replete (18 mM) and deficient (6 mM) nitrate supply. Each value is expressed as mean ± SD (*n* = 3).

To evaluate the potential of fucoxanthin production in *O. aurita*, growth experiments were conducted in the Ø 3 cm of bubble column photobioreactor with a nitrogen-replete (18 mM) and a nitrogen-limited (6 mM) culture media. The cultures were subjected to a low (100 µmol photons m^−2^ s^−1^) and a high (300 µmol photons m^−2^ s^−1^) light intensities. At the low light, *O. aurita* exhibited nearly identical growth with the two different concentrations of nitrogen, and the maximum biomass concentration (*ca.* 4 g L^−1^) of the cultures occurred on day 10 (Figure 1A). At the high light, more rapid growth occurred in the cultures containing the higher nitrogen concentration and the maximum biomass concentration of 6.36 g L^−1^ was obtained on day 10, which was about 50% higher than that (4.24 g L^−1^) in the low nitrogen cultures (Figure 1B).

At the low light, the fucoxanthin concentration in the low nitrogen cultures decreased from 16.71 mg g^−1^ to 6.34 mg g^−1^ during a 12-day cultivation. In the high nitrogen cultures, the pigment increased to 20.83 mg g^−1^ on day six and then gradually decreased to 15.70 mg g^−1^ at the end of the 12-day culture period (Figure 1C). At the high light, the changes in fucoxanthin concentration in both the low and high nitrogen cultures followed essentially the same trends of their counterparts under the low light with a slight difference being that the final pigment concentrations in the low and high nitrogen cultures were about half of that occurred in their counter parts under the low light (Figure 1D). 

Carreto and Catoggio found that the cellular fucoxanthin and chlorophyll contents in *Phaeodactylum tricornutum* decreased with the age of culture, accompanied by a slight increase in the diadinoxanthin content [17]. Unlike secondary carotenoids (e.g., β-carotene, astaxanthin), which play a role in preventing excess light energy from reaching the photosynthetic machinery, fucoxanthin act as a primary carotenoid, whereby transferring light energy to the photosynthetic reaction centers for photosynthesis [14]. Under stress conditions, changes in the organization of the photosynthetic apparatus (e.g., chloroplast fragmentation, degradation of thylakoid membrane) occur, chlorophyll *a* and other pigments involved in photosynthesis decrease, while the secondary carotenoids increase. These variations in pigment content might be as a quotient between photosynthetically active pigments and other functional pigments.

The fucoxanthin volumetric concentration and productivity of the microalga cultivated under the different conditions was compared (Table 1). The highest fucoxanthin volumetric concentration and productivity in the low nitrate supply and low light intensity cultures were 27.11 mg L^−1^ and 2.71 mg L^−1^ day^−1^, respectively, which increased to 76.73 mg L^−1^ and 7.67 mg L^−1^ day^−1^, respectively in the nitrate-replete cultures. However, further increasing light intensity from 100 to 300 µmol photons m^−2^ s^−1^ led to a considerable decrease of the fucoxanthin concentration in the cells, the biomass concentration had a significant enhancement under the high light intensity. The maximum fucoxanthin volumetric concentration of 79.56 mg L^−1^ was obtained with replete nitrate supply and high light intensity, resulting in a record high fucoxanthin volumetric productivity of 7.96 mg L^−1^ day^−1^.

**Table 1:** The fucoxanthin concentration, volumetric concentration and volumetric productivity of *O. aurita* cultivated in bubble column photobioreactors with replete (18 mM) and deficient (6 mM) nitrate supply under low (100 μmol photons m^−2^ s^−1^) and high (300 μmol photons m^−2^ s^−1^) light irradiation on day 10.

		Fucoxanthin		
Light intensity (μmol photons m^−2^ s^−1^)	Nitrate concentration (mM)	Concentration (mg g^−1^)	Volumetric concentration (mg L^−1^)	Volumetric productivity (mg L^−1^ day^−1^)
100	6	6.71	27.11	2.71
	18	18.14	76.73	7.67
300	6	4.28	18.15	1.82
	18	12.51	79.56	7.96

### 2.2. Mass Production Potential in the Pilot-Scale Flat Plate Photobioreactor

**Figure 2:**
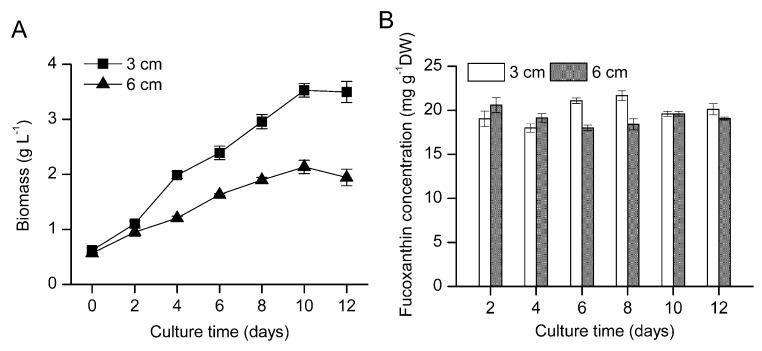
The growth profile (**A**) and fucoxanthin concentration (**B**) of *O. aurita* cultivated in the 3 cm (75 L) and 6 cm (150 L) flat plate photobioreactor under 300 μmol photons m^−2^ s^−1^ light intensity with replete (18 mM) nitrate supply. Each value is expressed as mean ± SD (*n* = 3).

To assess culture system scale-up feasibility, *O*. *aurita* was cultivated in a pilot-scale flat plate photobioreactor with replete (18 mM) nitrate supply under a high light intensity of 300 µmol photons m^−2^ s^−1^. Results showed that the light path exerted a strong influence on growth of this microalga. The maximum biomass concentration of 3.53 g L^−1^ was obtained in a 3 cm light-path photobioreactor (75 L volume) on day 10, which decreased to 2.14 g L^−1^ when the reactor light path increased to 6 cm (150 L volume) (Figure 2A). As indicated in Figure 2B, litter difference in fucoxanthin concentration was observed in the two light path photobioreactors, and the fucoxanthin concentration in the dry biomass was stabilized at a high level of 18.01~21.67 mg g^−1^. 

As a result, the fucoxanthin volumetric productivity of the 3 cm light path photobioreactor was greater than that obtained in the 6 cm light path one (Table 2). The fucoxanthin volumetric productivity in 3 cm light-path photobioreactor was comparable to that obtained in the smaller volume bubble column bioreactor, demonstrating the promising feasibility of *O*. *aurita* culture at scale. 

**Table 2:** The fucoxanthin concentration, volumetric concentration and volumetric productivity of *O. aurita* cultivated in 3 cm and 6 cm flat plate photobioreactors under 300 μmol photons m^−2^ s^−1^ light irradiation on day 10.

	Fucoxanthin		
Light path (cm)	Concentration (mg g^−1^)	Volumetric concentration (mg L^−1^)	Volumetric productivity (mg L^−1^ day^−1^)
3	19.59	69.15	6.92
6	19.61	41.97	4.20

Microalgae are considered as alternative sources for various bioactive compounds. Several carotenoids, such as astaxanthin, lutein, and β-carotene, have been commercially produced from microalgae. For example, the chlorophycean microalga *Muriellopsis* sp. has a high lutein volumetric concentration of *ca.* 35 mg L^−1^ [18]. Commercial production of natural β-carotene was obtained by mass cultivation of the microalga *D*. *salina* obtained a high β-carotene volumetric concentration almost of 150 mg L^−1^ [19]. Natural astaxanthin produced by *H*. *pluvialis* has applications in the aquaculture and nutraceutical markets [20]. Fucoxanthin is a major carotenoid present in brown seaweeds and diatoms, and is very effective in chemoprevention of cancer in animal studies [21]. Most of these studies were conducted with fucoxanthin isolated from macroalgae, and the fucoxanthin concentration from these macroalgae ranged from 0.02 to 0.58 mg g^−1^ in fresh samples and 0.01 to 1.01 mg g^−1^ in dried samples (Table 3). In contrast, the reported fucoxanthin concentration in microalgae ranges from 2.24 to 18.23 mg g^−1^, which is in one to three orders of magnitude greater than that found in macroalgae, indicative of the great potential of diatoms as a promising source of fucoxanthin for various commercial applications. The fucoxanthin concentration of 3.33–21.67 mg g^−1^ and the volumetric concentration of 18.15–79.56 mg L^−1^ obtained in the present study have set the records for algae-based fucoxanthin production.

**Table 3:** The fucoxanthin concentrations in the samples of different macroalgae and microalgae ^1^.

	Species	Fresh or dried	Fucoxanthin concentration (mg g^−1^)	References
Macroalgae	*Eisenia bicyclis*	Fresh	0.26	[7]^ 2^
	*Hizikia fusiformis*	Fresh	0.02	[5] ^2^
	*Laminaria japonica*	Fresh	0.19	[5] ^2^
	*Laminaria japonica*	Fresh	0.03	[22] ^2^
	*Petalonia binghamiae*	Fresh	0.43–0.58	[23] ^3^
	*Scytosiphon lomentaria*	Fresh	0.24–0.56	[23] ^3^
	*Sargassum fusiforme*	Dried	0.01	[22] ^2^
	*Sargassum binderib*	Dried	0.73	[24] ^2^
	*Sargassum duplicatum*	Dried	1.01	[24] ^2^
	*Sargassum plagyophyllum*	Dried	0.71	[25] ^2^
	*Turbinaria turbinata*	Dried	0.59	[25] ^2^
	*Undaria pinnatifida*	Dried	0.73	[22] ^2^
	*Undaria pinnatifida*	Fresh	0.11	[5] ^2^
Microalgae	*Chaetoceros gracilis*	Dried	2.24	[26] ^2^
	*Cylindrotheca closterium*	Dried	5.23	[27] ^2^
	*Isochrysis* aff. *Galbana*	Dried	18.23	[26] ^2^
	*Isochrysis galbana*	Dried	6.04	[26] ^2^
	*Phaeodactylum tricornutum*	Dried	8.55	[26] ^2^
	*Phaeodactylum tricornutum*	Dried	15.42–16.51	[7] ^2^
	*Nitzschia* sp.	Dried	4.92	[26] ^2^
	*Odontella aurita*	Dried	21.67	*In this study*

^1^ Fucoxanthin concentration in algal samples were expressed as mg g^−1^ dry (or fresh) weight of algal samples, unless special statement, fucoxanthin concentration represented all-*trans*-fucoxanthin concentration; ^2^ The fucoxanthin concentration was quantified by HPLC using a standard curve with purified fucoxanthin or standard chemical; ^3^ The fucoxanthin concentration was quantified by UV spectroscopy with E value (1%, 1 cm) of 1197 at 451 nm determined authentic fucoxanthin in MeOH-H_2_O (9:1).

### 2.3. Optimization of Fucoxanthin Extraction Conditions

**Figure 3:**
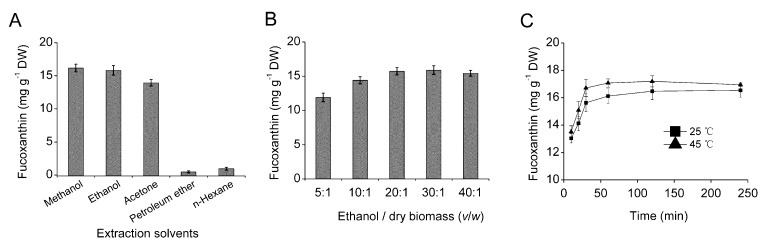
The effect of (**A**) solvent type, (**B**) ethanol/dry biomass ratio (v/w), and (**C**) extraction temperature and time on the extracted fucoxanthin concentration from freeze-dried *O. aurita*. Each value is expressed as mean ± SD (*n* = 3).

The limited availability of natural fucoxanthin to date is not only due to the lack of sufficient supply of fucoxanthin-rich biomass feedstock, but also poor extraction efficiency of fucoxanthin from biomass [5,26]. In order to determine the optimal conditions for fucoxanthin extraction, five conventional solvents were tested to determine the most suitable solvent for extraction of fucoxanthin from *O*. *aurita*. The results showed that fucoxanthin extraction efficiency was highly dependent on the solvent type. Fucoxanthin concentration extracted with methanol (16.18 mg g^−1^ DW) was the highest among the five tested solvents, followed by ethanol (15.83 mg g^−1^ DW) and acetone (13.93 mg g^−1^ DW). Petroleum ether and *n*-hexane did not effectively extract fucoxanthin from *O. aurita* (Figure 3A). The results were in accordance with findings reported by Kim *et al.* [7], who isolated fucoxanthin from the microalga *P*. *tricornutum* with different solvents, and the maximum fucoxanthin concentration (15.33 mg g^−1^) was obtained with ethanol, while acetone extracted approximately one third of that with ethanol. Although ethanol had slightly lower extraction efficiency than methanol, it exerts lower toxicity, and thus was selected as the most suitable extraction solvent in further research. 

Selecting a proper ratio of solvent to dry algal biomass (v/w) is important, as it may affect the quantity and quality of fucoxanthin. As shown in Figure 3B, up to 20:1 ethanol/dry biomass, the fucoxanthin extraction efficiency increased remarkably (11.90–15.74 mg g^−1^ DW) with increasing the ethanol to dry biomass ratio. When *O*. *aurita* dry biomass was treated with 30:1 ethanol/dry biomass, the fucoxanthin concentration increased slightly (15.90 mg g^−1^ DW), and further increasing of ethanol did not improve the fucoxanthin extraction efficiency. So, the ethanol/dry biomass ratio of 20:1 was sufficient for effective extraction of fucoxanthin from this microalga. 

To assess the effect of temperature and extraction time on the fucoxanthin extraction efficiency, *O*. *aurita* was extracted at 25 °C and 45 °C with different extraction time, *i.e.*, 10, 20, 30, 60, 120, and 240 min. As shown in Figure 3C, increasing the extraction temperature from 25 °C to 45 °C increased the extracted fucoxanthin concentration from 16.12 mg g^−1^ to 17.20 mg g^−1^ DW, attributable likely to the elevated temperature enhanced solubilization of photosynthetic membranes and release of fucoxanthin from fucoxanthin-Chl *a*, *c*-protein complexes [5,7]. The extracted fucoxanthin concentration was also a function of extraction time. At 45 °C, approximately 80% of fucoxanthin corresponding to 13.53 mg g^−1^ was extracted from algal biomass within the first 10 min, and the maximum fucoxanthin concentration was obtained at approximately 60 min. 

### 2.4. Purification and Identification of Fucoxanthin

**Figure 4:**
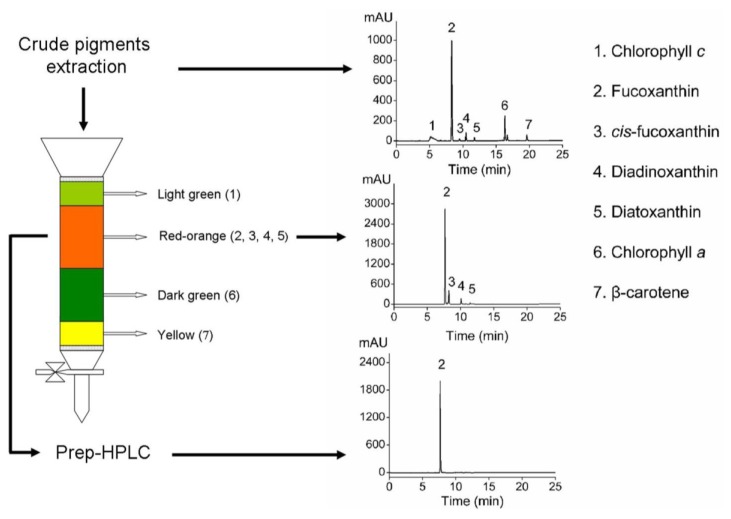
Isolation and purification fucoxanthin from crude pigments extraction of *O. aurita* through silica gel column chromatography and preparation high performance liquid chromatography (prep-HPLC). All fractions were analyzed by HPLC for qualitative.

Fucoxanthin was extracted and purified following the procedure illustrated in Figure 4. Firstly, pigments were extracted from freeze-dried algal powder according to the optimized extraction conditions. The pigment composition of crude extracts was analyzed by HPLC and the results showed that fucoxanthin, *cis*-fucoxanthin, diadinoxanthin, diatoxanthin, and β-carotene were the major carotenoids in this species, accompanied by chlorophyll *a*, *c* as its major chlorophyll pigments. The results were in accordance with the pigment profile previously reported in other diatoms. Stauber and Jeffrey analyzed the photosynthetic pigments of 51 species of marine diatoms, and found that all species contained chlorophyll *a* and *c*_2_ and β-carotene, fucoxanthin, diatoxanthin and diadinoxanthin [28].

The crude pigment extracts were subject to open silica gel column chromatography with *n*-hexane/acetone (6:4 solution; v/v) being the eluting solvent system, and all fractions were analyzed by HPLC. An orange-red colored fucoxanthin-rich fraction, which consisted of fucoxanthin, *cis*-fucoxanthin, diadinoxanthin, and diatoxanthin, was separated by the column. The purity of fucoxanthin in the mixture was identified as high as 86.7%. Further purification of fucoxanthin was carried out by a prep-HPLC, and pure fucoxanthin was collected. The purity of the fucoxanthin was >97% as determined by HPLC. 

**Figure 5:**
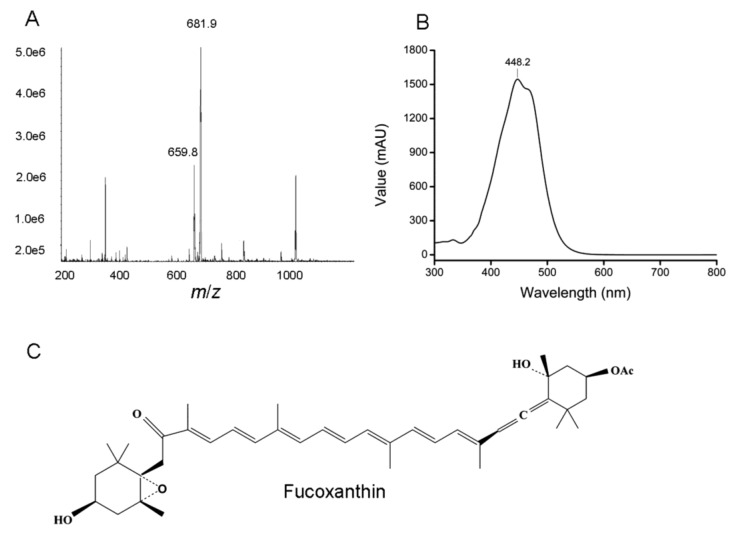
Identification of purified fucoxanthin from *O. aurita*. (**A**) The mass fragments; (**B**) UV-visible spectrum; (**C**) chemical structure of all-*trans* fucoxanthin.

The purified fucoxanthin was analyzed by LC-MS and NMR. The molecular mass spectrum corresponding to the molecular weight of fucoxanthin was identified based on the fragment pattern at *m*/*z* 659.8 and 681.9 corresponding to [M + H]^+^ and [M + Na]^+^, respectively (Figure 5A). The purified fucoxanthin showing the same retention time and UV-visible spectrum (λ_max_ at 448 nm) with the fucoxanthin standard confirmed the molecule identification of the pigment (Figure 5B). The purified fucoxanthin was subjected to NMR spectroscopy for its structural determination (Table 3). The complete assignments of the ^1^H and ^13^C NMR spectra of fucoxanthin revealed the signals assignable to polyene containing acetyl, conjugated ketone, olefinic methyl, two quaternary germinal oxygen methyls, two quaternary germinal dimethyls, and allene groups. The NMR data agreed well with the findings from the diatom *P*. *tricornutum* and haptophyte *Isochrysis* aff. *galbana* [7,29], suggesting that fucoxanthin is major in a all-*trans* form in *O*. *aurita* (Figure 5C).

### 2.5. Antioxidant Activity

Reactive oxygen species (ROS) formed under photooxidation stress can react with macromolecules like lipids and proteins leading to cellular damage. Antioxidants are substances that have the ability to reduce ROS and prevent macromolecules from oxidation [30]. 1,1-Dihpenyl-2-picrylhydrazyl (DPPH) and 2,2′-Azino-bis(3-ethylbenzthiazoline-6-sulfonic acid (ABTS) radicals were stable radical sources for evaluation of free radical-scavenging ability of various compounds [31].

**Figure 6:**
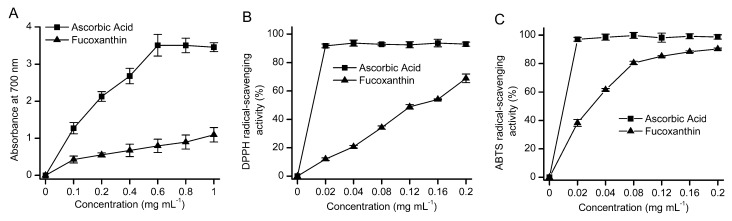
Antioxidant assays for the purified fucoxanthin from *O. aurita*. (**A**) Reducing power; (**B**) scavenging of DPPH radical; (**C**) scavenging of ABTS radical. Values were representative of three independent experiments.

To evaluate the antioxidant capacity of fucoxanthin purificated from *O. aurita*, DPPH and ABTS-based radical scavenging and reducing power assays were carried out. Ascorbic acid, a standard antioxidant, was used as a positive control (Figure 6). As the fucoxanthin concentration increased from 0.1 mg mL^−1^ to 1 mg mL^−1^, the reducing power of fucoxanthin increased in a concentration-dependent manner, thought the reducing capacity was somewhat lower than that of ascorbic acid (Figure 6A). The similar scavenging activity pattern was observed in the DPPH assay. The DPPH radical scavenging activity was linearly dependent on the fucoxanthin concentration; the effective concentration for 50% scavenging (EC_50_) was 0.14 mg mL^−1^ (Figure 6B). Fucoxanthin was an excellent scavenging agent to ABTS radicals. The ABTS radical scavenging rate increased in a concentration-dependent manner when fucoxanthin concentration increased from 0.02 mg mL^−1^ to 0.08 mg mL^−1^_,_ and the EC_50_ for ABTS radical was almost 0.03 mg mL^−1^. A plateau occurred at 0.2 mg mL^−1^ with a scavenging rate of 90.3%.

It was reported that the extracts of brown seaweed *Cystoseira hakodatensis* exhibited a strong DPPH radical scavenging activity, due largely to the presence of fucoxanthin [32]. Sachindra *et al.* [33] assessed the radical scavenging abilities of macroalgae-derived fucoxanthin and its two metabolites—fuxoxanthinol and halocynthiaxanthin—against DPPH, ABTS, hydroxyl radical, and superoxide radical, and suggested that fucoxanthin and fucoxanthinol exhibited higher than or similar activities to α-tocopherol.

## 3. Experimental Section

### 3.1. Organism and Culture Conditions

*O*. *aurita* was obtained from the Scandinavian Culture Collection of Algae and Protozoa at the University of Copenhagen and maintained in a modified L1 medium prepared from artificial seawater [34].

Culture experiments were conducted with three types of photobioreactors: Ø 3 × 60 cm bubble columns with a working volume of 320 mL and a flat plate photobioreactor measuring 3 cm depth, 240 cm length × 120 cm height with a working volume of 75 L, and a similar photobioreactor measuring 6 cm depth, 240 cm length × 120 cm height, with a working volume of 150 L. Cultures were carried out indoor at 25 ± 2 °C and aerated with compressed air containing 1% CO_2_. Continuous illumination was provided by a bank of cool white fluorescent lamps from one side of photobioreactor at an intensity of 100 or 300 µmol photons m^−2^ s^−1^. Light intensity was measured on the outer surface of photobioreactor with a dual radiation meter (Apogee DRM-FQ, Logan, UT, USA). 

### 3.2. Biomass Measurement

Ten milliliters (10 mL) culture was filtered through a pre-weighed GF/B filter paper and washed with ammonium formate. The filter paper with algal sample was dried in an oven at 105 °C overnight, and then weighed. The dry weight (DW) of sample was calculated by the difference in weights of the filter paper with and without algal sample.

### 3.3. Pigment Extraction and Analysis

Ten milligrams (10 mg) freeze-dried microalgal powder was extracted with 5 mL ethanol in conical centrifugation tube with a magnetic stirrer for 2 h in the dark. After extraction, the mixture was centrifuged for 5 min at 3500 rpm and supernatant was collected for pigments analysis by HPLC. Two hundred milligrams (200 mg) freeze-dried microalgal powder cultivated in the 3 cm flat plate photobioreactor on day 12 was used for the optimization of extraction conditions. Various solvents (methanol, ethanol, acetone, petroleum ether, and *n*-hexane), solvent volume to dry biomass weight ratio (5:1, 10:1, 20:1, 30:1, and 40:1), extraction temperature (25 °C and 45 °C) and time (10, 20, 30, 60, 120, and 240 min) were tested to determine the optimal conditions for fucoxanthin extraction. Every procedure was performed under dim light to prevent pigment degradation or photooxidation. All experiments were performed independently in triplicate. Each pigment extraction was filtered through a 0.2 μm Nylon membrane filter (Millipore, Billerica, MA, USA) before HPLC analysis.

### 3.4. Purification of Fucoxanthin

Based on the optimal extraction conditions developed above, 50 g freeze-dried microalgal powder produced from a 12-day culture of *O. aurita* in the 3 cm flat plate photobioreactor was extracted with 1 L ethanol at 45 °C for 1 h. The extracts were filtered and then concentrated in a rotary evaporator (Büchi R-205V805, Flawil, Switzerland) under vacuum conditions at 45 °C. The concentrated extracts were loaded onto a silica gel (200–300 mesh, Qingdao Haiyang Chemical Co., Ltd., Qingdao, China) packed into a glass column (2 × 30 cm) and equilibrated with a mixture of *n*-hexane:acetone (6:4), then eluted with the same solvent. The orange-red fucoxanthin-containing fraction was collected and concentrated into a small volume. Further purification of fucoxanthin was carried out by prep-HPLC. 

### 3.5. HPLC and LC-MS Analysis

Pigment analysis was performed on a Dionex model U-3000 HPLC (Dionex, Sunnyvale, CA, USA). A Kromasil C_18_ reverse phase column (5 μm particle size, 250 × 4.6 mm ID, Dionex, Sunnyvale, CA, USA) coupled with a C_18_ guard column (5 μm particle size, 15 × 4.6 mm ID) was used. The mobile phases and the elution program were adopted from our previous report [35]. A calibration curve (10–200 μg mL^−1^) was established for quantification of fucoxanthin by HPLC using a all-*trans*-fucoxanthin standard (Cayman Chemical, Ann Arbor, MI, USA). The amount of fucoxanthin was calculated from the peak area by the standard curve. Fucoxanthin concentration in the microalgal samples were expressed as mg g^−1^ dry weight of samples. Purified fucoxanthin was analyzed with an Agilent 1100 module (Agilent Technologies, Wilmington, DE, USA), coupled with an API 4000 Q-TRAP MS system (Applied Biosystems, Foster City, CA, USA). The mobile phase and gradient conditions were the same as that for the HPLC analysis. The MS conditions were set as follows: positive ions in the range from *m*/*z* 200–1000 were measured. An ion source voltage of 5.5 kV, a cone voltage of 60 V, a nebulizing gas of 30 psi, and a curtain gas of 10 psi were applied.

### 3.6. NMR Analysis

Purified fucoxanthin (10 mg) from Prep-HPLC was dissolved in 1 mL CD_3_OD and used for NMR spectroscopy. The ^1^H and ^13^C NMR signals were recorded on a Varian Inova 500 MHz NMR system (Vernon Hills, IL, USA) with a carbon enhanced cold probe (^1^H with 500 MHz, ^13^C with 126 MHz). Chemical shifts were adjusted with δ (ppm) referring to the solvents peaks δ_H_ 3.31 and δ_C_ 49.2 for CD_3_OD. Data were processed with the MestReNova program and compared with data in the literature. 

### 3.7. Assay for Antioxidant Activity

The reducing power of fucoxanthin was determined according to the method of Deng [36] with minor modification. Briefly, 1 mL ethanolic fucoxanthin solution (0.1–1 mg mL^−1^) was mixed with 0.2 mL 0.2 M sodium phosphate buffer (pH 6.6) and 1.5 mL 1% (w/v) potassium ferricyanide. The mixture was incubated at 50 °C for 20 min under water bath. Then 1 mL 10% (w/v) trichloroacetic acid was added. The resultant mixture was centrifuged for 10 min at 3500 rpm. Two milliliters (2 mL) supernatant was diluted with 3 mL distilled water and then mixed with 0.5 mL 0.3% (w/v) ferric chloride. The absorbance was measured at 700 nm against a blank. Ascorbic acid was taken as a positive control. The increased absorbance indicates an increased reducing power. 

The scavenging activity of DPPH radical was determined as describe by Sachindra *et al.* [33]. Briefly, 2 mL ethanolic fucoxanthin solution (0.02–0.2 mg mL^−1^) was mixed with 2 mL 0.16 mM ethanolic solution of DPPH. The mixture was shaken vigorously and incubated for 30 min at room temperature in the dark. The absorbance was measured at 517 nm. Ascorbic acid was taken as a positive control. The scavenging ability was calculated as: DPPH radical scavenging activity (%) = [1 − (A_1_ − A_2_)/A_0_] × 100, where A_0_ is the absorbance in the lack of fucoxanthin (using distilled water instead of fucoxanthin), A_1_ is the absorbance in the presence of fucoxanthin, and A_2_ is the absorbance of ethanolic fucoxanthin solution (using ethanol instead of DPPH).

The scavenging activity of ABTS radical was measured as described by Osman [37] with minor modifications. The pre-formed ABTS free radicals were generated by reacting 7 mM ABTS diammonium salt and 2.45 mM potassium persulphate overnight at room temperature in the dark. The solution was diluted with 95% (v/v) ethanol until the absorbance at 734 nm reaching 0.7 ± 0.01 units. Three milliliters (3 mL) diluted ABTS radical solution was added to 1 mL ethanolic fucoxanthin solution (0.02–0.2 mg mL^−1^). The mixture was incubated at 30 °C for 60 min in a water bath. The absorbance at 734 nm was measured. Ascorbic acid was taken as a positive control. The scavenging ability was calculated as: ABTS radical scavenging activity (%) = [(A_0_ − A)/A_0_] × 100, where A_0_ is the absorbance of the control reaction, and A is the absorbance of fucoxanthin solution.

## 4. Conclusions

The production of fucoxanthin from *O. aurita* is very attractive, with the maximum yield of 79.56 mg L^−1^ achieved in the bubble column photobioreactor. A comparable yield obtained in the scale-up flat plate photobioreactor confirmed the technical feasibility and scalability of *O. aurita*-based fucoxanthin production on a large-scale. Moreover, a rapid and effective procedure for extraction and purification fucoxanthin from the microalga was developed. The purified fucoxanthin was identified as all-*trans* fucoxanthin, and showed strong antioxidant properties. These results suggested that *O. aurita* may be a promising natural source of fucoxanthin for human health.
